# Supplementary material for: Are carbon emissions trading and green financial instruments synergistic? -Comprehensive quantitative research based on content analysis
Source: PLoS One. 2024 Mar 7;19(3):e0298601. doi: 10.1371/journal.pone.0298601 (PMC10919601; doi:10.1371/journal.pone.0298601)
Supplement: S1 Data — (ZIP) [file pone.0298601.s001.zip › Data/地级市Z_F工作报告.zip!/╣π╢1⁄2/╣π╓▌╩╨/└┤╫╘╣1⁄2╓┌║┼í╛┬φ┐╦╩2╛▌═°í┐_2008.docx]

各位代表：

　　我代表广州市人民政府，向大会作工作报告，请予审议，并请政协委员和其他列席人员提出意见。

　　一、2007年工作回顾

　　过去的一年，在省委、省政府和市委的正确领导下，市政府坚持以邓小平理论和“三个代表”重要思想为指导，深入贯彻落实科学发展观，认真贯彻党的十七大精神，围绕建设现代化大都市、构建和谐社会的目标，积极实施“富民强市、科教兴市、依法治市、可持续发展”战略，按照“科学发展、环境优先，关切民生、解决民忧”的工作思路，依靠全市人民，完成了市十三届人大一次会议确定的目标任务，各项工作取得新的成就。

　　（一）综合经济实力再上新台阶。经济平稳快速增长，结构不断优化，质量和效益大幅提高。去年全市实现地区生产总值7050.78亿元，同比增长14.5%；人均地区生产总值达9302美元，同比增长11.3%。源于广州地区的财政一般预算收入达2116亿元，同比增长22.4%，其中地方一般预算财政收入523.79亿元，同比增长22.6%。经济总量和财政收入都比2002年翻了一番多。全年完成工业总产值9870.57亿元，新增产值1758.17亿元，增长20%。汽车、石化、电子产品制造三大支柱产业总产值同比增长24.1%，其中汽车制造业增长37.4%。全市规模以上工业经济效益综合指数为220.93，同比提高27点，规模以上工业企业实现利润总额733.08亿元，同比增长36%。金融、物流、会展、商贸、信息、旅游等现代服务业发展势头良好，全年第三产业实现增加值4072.8亿元，同比增长14.1%，占地区生产总值的57.76%。金融业增加值280.47亿元，增长16.1%，企业境内外上市11家，数量为历史最多。白云国际机场旅客吞吐量达3095.51万人次，跃居内地机场第二位，跨入世界大型航空枢纽机场行列；广州港货物吞吐量达3.41亿吨，其中集装箱吞吐量达920.36万标箱，同比增长38.3%。全市批发零售业商品销售总额达10996.58亿元，社会消费品零售总额增长18.9%。旅游业总收入798.15亿元，同比增长13.9%，来广州旅游观光的客人突破1亿人次大关。开放型经济快速发展，广州地区进出口总值734.94亿美元，同比增长15.2%。其中，出口总值379.02亿美元，同比增长17%。实际使用外商直接投资32.86亿美元，同比增长12.4%，在广州投资的世界500强企业已达160个。民营经济增加值增长14%，占全市地区生产总值的34.24%，成为全市经济增长的重要力量。

　　（二）节能减排工作取得显著成效。启动创建循环经济试点城市活动，制订节能定额和节能标准，建立健全节能减排责任制，严格新建项目能耗环保审批，重点抓好110多家用能大户的节能工作。实施中心城区产业“退二进三”战略，加快淘汰落后生产力，引导企业在搬迁改造中实现技术升级。关闭水泥厂19家，淘汰落后产能533万吨。大力推进工业企业脱硫工程建设，全市累计形成9.8万吨/年的脱硫能力。各项节能减排指标控制良好，全市万元生产总值能耗下降4%，化学需氧量和二氧化硫排放量分别下降2.2%和8.8%，节约标煤约20万吨，节水300万吨。

　　（三）创新型城市建设迈出新步伐。制定了关于提高自主创新能力若干规定及配套政策。自主创新条件建设取得新进展，新增国家重点实验室3家，国家级和省级企业技术中心9家。一批骨干企业相继建立研发机构，以企业为主体的创新体制逐步形成，创新要素加速向企业集聚。大力发展高新技术产业，设立了“软件和动漫产业发展专项资金”，扎实推进软件、电子信息、生物医药和网游动漫等国家级高新技术产业基地建设，数字通信、平板显示器和生物医药等一批高科技创新项目建设投产，全市高新技术产品产值达2963.09亿元，同比增长28.7%，占工业总产值的比重达30.02%。积极创建国家知识产权示范城市，全市专利授权量增长33.2%。

　　大力推进政府管理创新，基本完成机关事业单位工资制度改革，初步建立起规范的事业单位津贴补贴制度，事业单位人员聘用制改革稳步推进。进一步规范国有资产营运监管，建立了国有资产损失问责制和企业经营预算管理制度。加快国有企业资本结构调整和重组步伐，积极推进企业改制上市，国有经济活力、竞争力和影响力进一步增强。

　　（四）城市环境建设与管理进一步加强。白云国际机场二期扩建工程按计划推进，南沙港区二期4个10万吨泊位建成使用，广州铁路新客站建设进展顺利。7条在建地铁线路及街北高速等一批高、快速道路建设全面推进。收购东南西环高速路并停止收费，优化了道路交通网络功能，节约了土地并缓解了交通压力。白云国际会议中心建成投入使用，广州国际会展中心二期完成主体工程建设。城市供水、供电和公交等市政公共服务设施不断完善。饮用净水服务人口达216.54万人，天然气置换已完成22.3万户。新建和改造港湾式公交站154座。为内环路沿线民居、医院、学校等建筑共安装67478平方米隔声窗，建成总长18公里的隔声屏障，环境噪声平均值下降0.2分贝。广州亚运城及各项亚运场馆工程和配套建设全面展开。人民防空体系进一步完善。“青山绿地、蓝天碧水”二期工程取得阶段性成果。林区林带及城市绿地系统加快建设，全市新增绿地28.46平方公里，239万亩生态公益林得到有效保护。城市水环境治理力度加大，沙河涌等7条河涌综合整治取得阶段性成效；新铺设截污管线136公里，新建的4个污水处理厂主体工程已完成，全市生活污水日处理能力达191.8万吨，中心城区生活污水处理率达82.34%。大型环卫基础设施不断完善，全市生活垃圾实现日产日清，无害化处理率达82.8%。新建和改建公厕130座，全市环卫公厕全部免费开放。加大饮用水源保护力度，特别是开工建设白云湖和推进实施西江引水工程，对改善我市水环境和市民饮水质量，保证我市水资源战略安全具有重大意义。综合整治大气环境污染，严格实施机动车排放国Ⅲ标准；逐步推进机动车环保标志管理；中心城区全面禁行摩托车；全市85%的公交车和100%的出租车已使用清洁能源。继续开展餐饮业油烟污染整治工作，强化对各类污染源的监管。空气质量持续改善，稳定保持在国家二级标准以上。动员全市力量，推进创建国家卫生城市工作，取得了突破性成果，顺利通过国家爱卫会的暗检和技术评估。认真贯彻国务院和省政府关于全面开展产品质量、食品安全专项整治的部署，强化对食品、药品和重点企业、重点区域、重点产品的监管，大力整顿和规范市场秩序，较好地完成了专项整治任务，保证了产品质量和食品安全。

　　（五）“三农”工作取得新成绩。各项支农惠农措施得到落实，市财政共安排涉农资金37.77 亿元，比上年增长15.3%。全年实现农业总产值285.4亿元，同比增长5.6%；农村居民人均纯收入8613元，同比增长10.6%。都市型农业发展迅速，农业现代化、产业化经营水平不断提高。对15.69万亩农田、2.1万亩鱼塘进行标准化改造。“万村千乡”市场工程全面推进，日用消费品农家店行政村、镇覆盖率均达100%，提前三年完成省下达的任务，为农村提供了7000多个就业岗位。对生猪生产给予补贴。免费对7000名农民进行农业科技培训。继续推进农村基础教育工程建设，农村中小学办学条件得到改善。农村义务教育阶段学生全部免学杂费、课本费，继续实行“持证”困难家庭义务教育阶段学生免书杂费和寄宿生补助生活费政策。投入超过1亿元，改造改建4所中心镇医院、20所镇卫生院和433所村卫生站，区（县级市）、镇、村三级卫生服务网络进一步完善。新型农村合作医疗制度不断完善，参合率达99.4%。进一步加大农村富余劳动力转移就业培训力度，共完成5.6万名农村劳动力转移就业。全市中心镇完成了总体规划编制，全面开展了行政村的规划编制工作。按照社会主义新农村建设要求，加快实施“两规、三清、四有、五通”工程，730条村开展了村容村貌整治，农村面貌和农民生产生活条件进一步改善。城乡水利防灾减灾工程建设任务累计完成90%，防御水旱灾难能力进一步增强。

　　（六）各项社会事业全面推进。市本级共投入教育事业经费29.39亿元，占年度一般预算支出15.3%。全市12个区、县级市全部创建成为省教育强区（县级市），我市顺利通过省教育强市督导验收，教育整体实力和水平迈上新台阶。义务教育规范化学校建设取得新进展，首批25所创建国家级示范性普通高中学校已通过省的初期督导验收，示范带动效应逐步形成。中等职业学校完成布局调整。市属高校坚持内涵发展，教学质量不断提高。推进依法落实教师待遇工作，市属各类学校、教育机构教师待遇进一步提高。文化建设不断加强，以“构建和谐社会，创建美好生活”为主题的系列活动广泛开展。文化事业和文化产业繁荣兴旺，杂技剧《西游记》和音乐剧《星》在第八届中国艺术节上双获文华大奖。新闻出版和广播电视事业取得新进步，文化市场执法成效明显。群众体育更为普及，竞技体育成绩突出，体育产业稳步发展。我市在第六届全国城市运动会上获奖牌和总分两项第一，成功举办40多项国内和国际重大赛事，开展了60项全市性大型群众体育活动。启动亚运城市行动计划，第十六届亚运会筹备工作扎实推进。民族宗教工作在促进经济社会发展中发挥积极作用。成功承办第八届全国少数民族传统体育运动会，展示了广州的良好形象，为促进民族团结作出了重要贡献。加强了流动人口的计生管理服务，开展了出生缺陷干预，全面完成了省下达的人口计划。社会救助、社会福利、救灾救济、慈善捐助、优抚安置、妇女儿童、老龄工作、残疾人事业全面发展。创建国家信息化示范城市工作取得积极进展，城市信息化综合指数达87%。对口扶贫工作力度加大，泛珠三角省会城市合作取得新进展。“双拥”工作、国防教育和后备力量建设进一步加强，广州连续六次被命名为“全国双拥模范城”。反走私和海防建设深入推进。外事、侨务工作成绩突出，国际友好城市达20个，我市行政首长当选世界城市和地方政府联合组织联合主席，进一步扩大了广州的影响力。统计、参事、文史、档案、保密、修志、气象、科普等工作有新的进步。

　　（七）民生福利不断改善。高度重视解决民生问题，市委、市政府制定并全面实施“66条”惠民政策措施，取得阶段性成效。大力推进创建充分就业社区工作，建立了促进高校毕业生就业公共服务机制，实施广州生源特困高校毕业生就业扶助制度，就业形势保持稳定。2007年末，城镇登记失业率为2.23%，城镇失业人员就业率为70%。社会保障体系不断完善，保障覆盖面进一步扩大，保障水平稳步提高。调整提高了全市50多万名企业退休人员月基本养老金和生活补贴，人均增加253元，同比提高22%。加快解决社保历史遗留问题，在全国率先将早期离开国企人员纳入养老保险体系。积极推进农转居人员和城市规划区内全征地农民参加基本养老保险工作，全市已有17.96万人参保。去年市本级安排0.76亿元解决国有困难企业职工参加基本医疗保险问题，安排1.89亿元资助社会申办退休人员缴交过渡性医疗保险金。农民工参加工伤保险人数达162万人，同比增长57%。将非本市城镇户籍职工纳入本市生育保险范围。构建和谐劳动关系取得明显成效。先后六次向低保和低收入家庭发放临时性生活补贴。维护市场价格秩序，城市居民消费价格指数上涨3.4%，低于全国4.5%和全省3.7%的水平。全面提高农村低保标准和五保供养水平，农村低保金月人均增至202元，提高12%。社区居家养老服务工作和“平安钟”呼援系统建设全面展开。262个社区获得省“六好”平安和谐社区称号。积极构建服务价位低、覆盖面广的城市公共卫生体系。制定出台了《广州市发展城市社区卫生服务的实施意见》和配套政策，建立了政府对社区公共卫生和基本医疗的补偿机制和大型医院对口帮扶社区卫生服务机构制度，启动了社区卫生服务机构药品统一配送试点工作，市区两级筹资7.62亿元，对64所社区卫生服务中心进行建设和改造。疾病防控工作成效显著，突发公共卫生事件监测、预警和应急处置能力不断提高。卫生监督执法逐步加强。中医药强市建设稳步推进。高度重视解决群众住房问题，制定了完善住房保障制度、抑制房价过快增长的七条措施。加快建设由廉租房、经济适用房、限价商品房和商品房构成的多层次住房保障和供应体系，加大住宅建设土地供应量，依法收回闲置土地，全面整顿房地产市场，有效缓解了住房供需矛盾，房价过快增长势头明显放缓。去年底已全部解决了5643户2005年登记在册、人均居住面积10平方米以下的双特困户住房问题。全市2004年在册的91.1万平方米危破房改造已完成90.8%。完成21项排水改造工程，解决了群众反映突出的68个地段“水浸街”的问题。继续加强社会治安综合治理，开展严打整治斗争，重点打击“两抢一盗”和黑恶势力犯罪，大力整治重点地区和突出治安问题。不断完善社会治安防控体系，治安视频系统基本覆盖市区范围。加强对流动人员、外籍人员的服务管理工作，出租屋安全隐患大幅减少。全年刑事案件同比下降15.8%，其中“两抢”和盗窃案件分别下降37.3%和12.8%。流花火车站广场等重点地区的治安整治工作取得突破性进展，群众对社会治安的满意度明显提升。加强了人民调解和人民内部矛盾排查调处工作，进一步扩大法律援助覆盖面，法律服务、基层建设等各项司法行政工作取得新进展。城市应急机制逐步完善，妥善处置了禽流感等突发疫情。深入开展安全生产隐患排查治理专项行动，安全生产形势平稳趋好，事故起数和死亡人数同比分别下降14.1%和5.3%，连续四年没有发生重特大火灾事故，森林山火面积同比下降78.8%。加快发展公共交通，新开通公交中（小）巴、水上公交试验线和常规公交接驳地铁专线共60条，积极推进公交地铁票价优惠改革，抓紧中心城区公共停车设施建设，方便了群众出行。人民生活水平逐步提高，去年，全市职工年人均工资达40187元，同比增长10.6%，城镇居民年人均可支配收入22469元，同比增长13.2%。

　　（八）民主法制和政府自身建设稳步推进。坚持向人大报告工作，认真执行人大及其常委会决议，自觉接受人大及其常委会和人民政协、各民主党派及社会各方面的监督。认真办理人大代表建议和政协委员提案，办复率达100%，办理质量和效率逐步提高。创新立法工作方法和机制，扩大公民有序参与行政立法工作，完成地方性法规草案4项，审议通过政府规章14项，清理市政府规章145件，废止、修改市政府规章74项。落实执法责任制，完善行政复议工作机制。深化行政审批制度改革，市政务服务中心加快建设。积极实施多种形式的公务员知识和技能培训，公务员综合素质有新的提高。稳步推进法制宣传教育，扎实开展反腐倡廉工作，强化执法监察、效能监察、廉政监察和预算执行审计，加大查处大案要案力度。认真开展以“为民、务实、清廉”为主题的机关作风建设年活动，行政效能有新的提高。

　　各位代表，过去的一年，广州改革开放和现代化建设取得新成绩，是全市人民和社会各界大力支持、共同努力的结果。在此，我代表广州市人民政府，向全市人民、驻穗人民解放军和武警部队官兵表示崇高的敬意！向关心和支持广州建设的港澳台同胞、海外侨胞和国际友人表示衷心的感谢！

　　在充分肯定成绩的同时，必须清醒看到，我市经济社会发展还面临不少问题和困难，主要是：粗放型经济发展方式尚未根本转变，结构调整的任务仍然比较艰巨；节能减排任重道远，环境保护的压力仍然比较大；自主创新能力和产业竞争力还不强；城乡发展和区域经济发展不平衡，城乡居民的生活水平和质量有待进一步提高；社会管理存在薄弱环节，公共服务体系还不够完善；解决事关群众切身利益问题与市民要求还有差距。我们务必高度重视解决这些问题。

　　二、2008年工作安排

　　2008年是我市全面贯彻落实党的十七大精神，加快推动经济社会转入科学发展轨道的关键一年。当前，经济全球化趋势进一步增强，我国改革开放步伐和工业化、现代化进程不断加快。广州的发展既面临前所未有的战略机遇，也面对许多困难与挑战。我们要坚决贯彻落实党中央、国务院和省委、省政府的各项决策部署，以世界眼光，在全省发展的大局中谋划广州新的发展，努力在全省新一轮思想大解放、实践科学发展观上闯出新路子、迈出新步伐，当好排头兵。

　　今年政府工作的总体要求是：全面贯彻党的十七大精神，高举中国特色社会主义伟大旗帜，坚持以邓小平理论和“三个代表”重要思想为指导，深入贯彻落实科学发展观，继续解放思想，围绕全面建设小康社会奋斗目标的新要求，抓住加快转变经济发展方式和完善社会主义市场经济体制两个关键，大力调整优化经济结构，大力推进改革开放和自主创新，大力加强节能减排和生态环境保护，大力促进城乡统筹发展，大力加强以改善民生为重点的社会建设，推动经济社会转入科学发展的轨道。

　　今年经济社会发展主要预期目标是：地区生产总值增长12%；全社会固定资产投资增长10%；社会消费品零售总额增长15%；外贸出口增长10%；实际使用外商直接投资增长9%；万元地区生产总值能耗降低4.35%；化学需氧量和二氧化硫排放量分别下降0.9%和10.4%；城市居民人均可支配收入增长10%；农村居民人均纯收入增长9%；居民消费价格涨幅控制在4%以内；城镇登记失业率控制在3.5%以内；人口自然增长率控制在5.45％。以内。重点抓好以下八项工作：

　　（一）坚持稳中求进、好字优先，促进经济又好又快发展。

　　从实施城市发展战略的高度推动区域发展。根据人口分布、资源环境承载能力和发展基础、发展潜力，进一步明确不同区域的功能定位，优化功能区和生产力空间布局，促进城市总体规划、土地利用总体规划与经济社会发展相衔接。加快推动东部、南部和北部三条产业带发展，增强高新技术和先进制造业集聚发展效应；充分发挥广州开发区和南沙开发区两个国家级开发区的示范辐射作用，带动周边区域加快发展；充分利用中心城区的独特优势和有利条件，突出发展总部经济和现代服务业，强化中心城区的核心作用和综合服务功能；推动番禺、花都发展保持良好势头，进一步提高发展质量与效益；加大对从化发展的扶持力度，增大其经济总量；推动增城加快发展，开辟广州新的重要发展区域，培育新的经济增长极。逐步形成功能定位清晰、区域良性互动、基本公共服务和人民生活水平差距趋向缩小的区域协调发展格局。

　　从改善城市环境的战略全局调整优化产业结构。着眼构建现代产业体系，在继续壮大汽车、石化和电子产品制造三大支柱产业的同时，积极吸引符合环保要求、发展前景好、处于产业链高端的先进制造业项目落户广州，促进工业向高科技、高集聚、高附加值发展。加快发展装备制造业，抓紧推进一批重大生产力项目建设。广泛运用先进适用技术改造提升传统优势产业，大力推进信息化与工业化相融合。实施以质取胜和名牌带动战略，进一步提高广州产品质量水平。制定政策措施，加快推进中心城区产业“退二进三”工作，逐步搬迁改造对环境影响大、资源耗用多的工业项目，腾出空间和环境容量发展具有可持续发展潜力的产业，使经济发展建立在节约资源和保护环境的基础上，实现工业质的飞跃，促进产业升级和城市环境优化。

　　从提高城市综合服务水平的高度加快发展第三产业。把发展第三产业、尤其是现代服务业放在更加突出的位置。一是大力发展金融业。加快市属金融机构改革重组步伐，完善金融产业政策体系，优化金融发展环境，吸引国内外金融机构和后台服务中心落户广州。充分利用资本市场，推动更多的企业上市。建设国家级区域性产权交易平台，探索发展债券市场。二是加快发展物流业。发挥空港、海港和区域铁路枢纽优势，推进国际物流园区建设。整合城区物流市场，扩大发展规模，提升发展水平。三是加快发展会展业。进一步优化会展业布局，做强现有会展项目，加快引入国际知名会展品牌，促进会展业市场化、产业化和国际化，培育成为新的支柱产业。四是加快发展商贸业。发挥广州“千年商都”的优势，培育主业突出、核心竞争力强的大型连锁企业集团，按照现代展贸市场的模式，推进批发市场升级改造，促进商贸经济的繁荣兴旺。五是优化发展饮食服务业。加快餐饮配套设施建设，打造特色鲜明的美食街，进一步提高“食在广州”的知名度和“美食之都”的品牌效应。六是加快发展旅游、文化和体育产业。打好“亚运”牌，发展“亚运”经济，举办更多的国际性体育赛事和文化交流活动，开发更多旅游品牌和旅游产品，吸引更多的中外客人到广州旅游和消费，不断提高广州国际知名度。

　　从增强城市发展协调性的要求统筹发展内外源经济。加快转变外贸发展方式，优化进出口结构，引导企业扩大技术含量高和具有自主知识产权的产品出口。大力发展服务贸易，积极拓展国际服务外包。继续加大招商引资力度，不断提高利用外资质量。加快发展总部经济，吸引更多的跨国公司和国内优势企业在广州设立地区总部或研发、运营和采购中心。加快实施“走出去”战略，鼓励和支持有实力的企业创新对外投资和合作方式，在生产、销售和研发等方面实施国际化经营。落实和完善鼓励支持民营经济发展的政策措施，支持民营资本进入公用事业、基础设施和金融服务业等行业。培育一批前景好和竞争力强的民营工业企业，推动民营工业优化结构，加快发展。

　　加大节能减排力度。增强全社会节约资源和保护环境意识。建立和完善节能减排统计体系、监测体系和考核体系。抓好全国首次污染源普查工作。继续推进重点领域和重点企业节能降耗技术改造，严格控制高能耗、高排放项目的立项审批，淘汰落后产能。突出抓好重点项目、重点工程、重点工业企业节能和减排达标监管工作。提高资源综合开发和循环利用效率。推进环境资源价格改革，建立健全环保价格体系，利用价格杠杆促进环境保护和资源节约。抓紧制定和启动新能源计划，大力发展和鼓励使用太阳能等可再生能源及替代能源。逐步关闭常规小火电发电机组，大力推进脱硫工程，坚决实现今年二氧化硫和化学需氧量减排控制目标。

　　（二）推进自主创新和高新技术产业发展，加快经济发展方式向创新驱动转型。

　　加快发展高新技术产业。市委、市政府出台关于大力推进自主创新加快高新技术产业发展的决定和配套措施，紧紧围绕重点领域和产业战略发展方向，加大发展高新技术产业的扶持和投入力度。重点发展电子产品制造业、生物产业、信息服务业和数控装备制造业，着力发展新材料、环保新能源、软件和创意产业，大力推进数字家庭建设。按照建设国家创新型科技园区的目标，高标准规划建设广州科学城、天河软件园、国际生物岛等高新技术产业园区，强化产业集聚效应。加快推进国家级电子信息、软件、网游动漫及生物四大产业基地建设，积极申报建设国家特色新材料产业基地，培育一批自主创新能力强、具有领先优势、发展前景好的高新技术企业和产业集群，尽快做大做强，使高新技术产业在我市现代产业体系中占据重要地位，整体提升经济发展后劲和竞争力。

　　加快完善自主创新体系。围绕建设创新型城市目标，强化企业自主创新的主体地位，鼓励企业建立工程研发中心和技术中心，加大研发和技改投入。支持企业与高校和科研院所建立战略合作关系，建设产学研结合的创新体系。积极为中小企业技术创新搭建服务平台，建立健全多元化、多渠道投融资体系。规划和建设一批共性和关键技术创新平台，支持和培育一批国家级和省级重点实验室，大力建设科技企业孵化器。吸引国内外研发机构落户广州，建设具有国际水平的研发基地。深入开展创建国家知识产权示范城市活动，依法保护智力成果，营造有利于自主创新的法制和市场环境。

　　加强创新人才的培养和引进。进一步落实科教兴市和人才强市战略，完善发现、培养、引进、使用、集聚优秀科技人才的体制机制和政策环境，为广州建设创新型城市提供人才保证和智力支持。以重点产业、重点学科、重大科研和工程项目为依托，培养造就一批科技领军人物和科技骨干。加快引进掌握核心技术和关键技术的高层次人才，以及文化、经济和社会领域的各类人才。继续办好中国留学人员广州科技交流会，实施“万名海外人才集聚工程”，完善优惠政策，吸引更多的一流科技创新人才和管理人才来广州创业发展。

　　（三）大力建设生态文明，进一步提高可持续发展的承载力。

　　调整完善城市规划布局。贯彻《城乡规划法》，按照广州城市发展战略，从增强城市发展承载力和优化城市空间布局、产业布局出发，认真做好功能区规划、新一轮城市总体规划和土地利用总体规划编制工作。正确处理人口资源环境关系，节约集约使用土地。在继续抓好广州铁路新客站、萝岗新城、南沙、空港新区、从化新温泉等区域规划建设的同时，突出抓好中心城区四个重点区域的规划建设：一是完善以会展经济为核心的琶洲地区及作为珠江新城延伸区的员村地区的规划和建设；二是加快城市新中轴线区域和珠江新城的建设，同时做好电视塔以南新中轴线地区的规划工作；三是研究完善白鹅潭经济圈和芳村大道等地区的规划功能和布局，逐步搬迁工业企业，大力发展第三产业；四是加快完善白云新城及周边地区的详细规划，打造新的商贸文化居住区。

　　继续推进城市基础设施建设。重点推进白云国际机场二期扩建工程、南沙港三期和出海航道三期疏浚工程，协调广州铁路新客站建设，提升海陆空交通枢纽承载能力。加快推进地铁和城市高快速道路系统建设，进一步完善交通网络。加快新电视塔、大剧院等标志性建筑建设。继续推进旧城区和“城中村”改造，努力改善人居环境。

　　进一步加强城市生态体系建设。继续推进“青山绿地、蓝天碧水”二期工程。实施森林围城计划，加强矿山地质生态环境恢复和城区绿化、林区林带建设。保护好万亩果林，逐步改造、建设成为果树生态公园。绿化美化城市主要出入口、主干道沿线、亚运场馆周边等重点地段，推进主要道路中间绿化隔离带建设，构建广覆盖、多层次的城市森林绿地系统，巩固国家园林城市创建成果，创建国家森林城市。继续抓好公交车、出租车使用清洁能源工作，对在用机动车全面实行环保达标管理，制定限制高排放车辆行驶的措施。严格控制工业废气和饮食服务业油烟污染。积极开展防治灰霾天气研究。加快环卫基础设施建设，提高垃圾处理能力。加大水系治理力度，强化饮用水源保护。继续推进城市污水处理系统建设，全面展开河涌综合整治工程，力争到2010年把市内八区主要河涌整治好。加快推进白云湖和西江引水工程建设，确保市民饮用水质量和我市水资源战略安全。

　　着力提高城市管理水平。认真总结三年城市管理年活动经验，加快建立城市管理长效机制，有效整合管理资源，促进城市管理制度化、规范化。巩固扩大“创卫”成果，建立环境保洁长效机制，毫不松懈地抓好市容环境卫生管理，努力实现“创卫”目标。健全食品药品监管，筹建市食品安全动态实时监控中心，规划建设广州食品物流配送基地，建立产品质量、食品安全管理的长效机制，确保产品质量和食品安全。继续加强出租屋和流动人员、外籍人员服务管理，进一步提高服务效能和管理水平。

　　（四）以实现农业发展、农民增收为根本，推进社会主义新农村建设。

　　大力发展现代农业。高标准建设都市型现代农业示范区、农产品基地和“专业镇”、“专业村”，完成15万亩农田、鱼塘标准化建设任务，提高农业规模化、集约化、产业化水平。推进科技兴农，着力发展特色农业和农产品加工，提高农业综合生产能力和经济效益。大力培植农业优势产业，扶持农业龙头企业发展，积极发展农业专业合作社和农村服务组织。开展政策性农业保险试点工作，建立健全现代农业保障体系。推进农业标准化，完善重大动植物疫情监测和预警机制，保证农业生产安全和农产品质量安全。

　　拓宽农民增收渠道。加大“三农”投入，落实支农惠农政策，形成农业增效、农民增收的良性互动机制。加强农业经贸合作和农产品市场开发建设，大力开发农业观光休闲旅游产品。鼓励农民自主创业，广辟农民外出务工经商渠道。大力发展农村二、三产业，继续加强农民转移就业技能培训，促进农村富余劳动力转移就业。加快发展农村职业教育，实施新型农民科技培训工程，增强农民文化科技素质和就业创业能力，促进农民持续增收。

　　加强农业基础设施建设。加快完善中心镇基础设施，增强中心镇人口和产业集聚能力，提高城镇化水平。进一步扩大社会主义新农村建设试点覆盖面，着力解决制约农业生产、农村生态、农民生活的基础设施问题。继续推进“万村千乡”市场工程。抓好农村环境综合整治，建设生态文明新农村。切实保障农民土地权益。加强农田水利基本设施建设，提高农业抵御自然风险能力。加大对从化以及北部6个山区镇的帮扶力度，增强山区镇自我发展能力。

　　推进农村社会事业建设。加快农村卫生服务体系建设，增强农村基本医疗服务能力。按照二级综合性医院规模推进中心镇医院建设，完成村卫生站新建和改建任务，加强对现有农村卫生技术人员的培训，做好对山区镇卫生院的帮扶工作。完善新型农村合作医疗制度，进一步提高参合率和筹资水平。落实农村免费义务教育政策，推动义务教育均衡发展。继续抓好农村中小学布局调整，建立农村义务教育校舍维修长效机制，改善办学条件，提升办学水平。探索建立农村养老保险制度。发展农村文化事业，改善农村文化体育活动条件。优化农村客运班车线路，方便农民出行。加强农村基层组织建设，健全村务公开和农村民主管理制度，抓好村民委员会换届选举工作，保持农村社会和谐稳定。

　　（五）深化经济领域和行政管理体制改革，为现代化大都市建设提供动力和体制保障。

　　大力推进新一轮国企改革。以深化公司治理机制改革为重点，着力深化公司制股份制改革，实现国有企业产权多元化。积极支持国有企业改制上市。继续推进国有经济的优化和重组，推动国有资本向优势产业、优势企业和优势产品集中，不断提高国有经济的活力、竞争力和带动力。进一步完善国有资产监督管理体制，逐步将全市经营性国有资产纳入统一监管，理顺国有企业领导人员管理体制，探索建立长效激励和约束机制，建立健全国有资本经营预算制度。

　　进一步推进行政管理体制改革。适应城市发展的新情况、新特点，完善“两级政府、三级管理、四级网络”城市管理体制，按照重心下移、权责一致、能放则放的原则，精简放权，扩大区一级管理权限。积极推进投融资体制改革，引导社会资金参与经营性公益事业和城市基础设施建设。推进水务体制改革，实现政府对水务的统一规划、建设和管理。加快建设市政务服务中心，推进政务公开和电子政务建设。深化公共财政体制改革，提高财政资金使用效益。继续精简和规范行政审批，提高政务服务效率。推进事业单位人事制度改革，完善岗位设置，健全岗位管理。探索建立公务员考核体系和政府绩效考核体系。

　　（六）大力推进文化和社会建设，促进各项社会事业加快发展。

　　加强文化建设。着眼增强城市文化软实力，大力建设社会主义核心价值体系，积极推进社会主义荣辱观教育、现代公民教育和未成年人思想道德教育，广泛开展城乡社会志愿服务活动和各类社会公益活动，弘扬民族精神和时代精神。大力推进理论创新，繁荣发展哲学和社会科学。深入开展创建全国文明城市活动，努力创建文明示范村、文明社区、文明行业和文明单位，不断提高市民文明素质。弘扬科学精神，普及科学知识。推进文化创新，深化文化体制改革，构建公共文化服务体系，培育文化人才和文化品牌。加强文化市场管理和网络文化建设，繁荣文艺创作，提高新闻出版、广播影视和版权工作的质量与水平，促进文化事业和文化产业全面发展。积极开展对外文化交流，创办有国际影响力的文化活动。高标准推进重点文化设施建设，加强对文物和非物质文化遗产的保护和利用，进一步彰显历史文化名城风采。

　　优先发展教育事业。巩固和扩大创建省教育强市成果，促进我市教育在新的起点上实现新的发展。加快义务教育规范化学校建设，促进教育均衡发展，逐步缩小城乡之间、区域之间、校际之间的办学差距。更加注重教育的普惠性，促进教育公平，全面实施城区免费义务教育，逐步解决进城务工农民子女接受义务教育问题。充分发挥示范性普通高中的作用，促进普通高中优质、协调发展。重视学前教育，关心特殊教育。大力发展职业教育，提高技能型人才的培养水平。推动市属高校进一步改善办学条件，提高教育教学质量。加强对民办学校的扶持和管理。加强教师队伍建设。全面实施素质教育，增强学生体质，提升学生的创新精神和实践能力。建立健全扶贫助学机制，保障贫困家庭子女接受教育的权利。

　　加快发展体育和其他社会事业。加强公共体育设施建设与管理，深入开展全民健身活动，提高竞技体育水平。全力办好2008年世界乒乓球团体锦标赛和其他 国际性、全国性体育大赛。千方百计稳定低生育水平，加强流动人口计划生育管理和服务，综合治理出生人口性别比偏高问题，提高计生工作水平和出生人口素质。高度重视妇女儿童工作，依法维护妇女儿童合法权益。积极主动做好新形势下民族和宗教工作，进一步促进民族关系、宗教关系的和谐。继续加强外事、港澳台和侨务工作，扩大国际交流与合作。做好第二次全国经济普查工作，进一步提升统计服务水平。增强国防观念，搞好国防动员和后备力量建设，推进“双拥”工作创新发展。全面落实优抚政策，认真做好军转干部、军队离退休干部和复退军人的安置工作。进一步做好档案、保密、修志、参事、文史等工作。

　　全力做好亚运会筹备工作。坚持政府主导与市场运作相结合、办会与后续利用相结合，积极推进亚运城和运动场馆及配套设施建设，带动周边地区改善环境，促进发展。组织实施亚运城市行动计划，动员、整合全市资源，全面提升各项工作水平，确保亚运会办出“中国特色，广东风格，广州风采”。

　　（七）着力改善民生，切实实现好、维护好、发展好广大人民群众的根本利益。

　　努力实现发展成果由人民共享。继续抓好市委、市政府“66条”惠民政策措施的贯彻落实，进一步加大公共财政对民生的投入，确保当年财政超收的80%以上用于发展社会事业和改善民生，不断优化公共资源配置，加强公共设施建设，增强政府公共产品和公共服务的供给能力。着力改善收入分配关系，加快建立健全包括公务员、事业单位和各类企业职工在内的工资正常增长机制，提高企业职工最低工资标准，进一步提高人民生活水平。

　　实施扩大就业发展战略。继续完善覆盖城乡的就业服务体系，全面推进创建充分就业社区工作，不断拓宽就业渠道，健全面向所有困难群众的就业援助制度，及时帮助零就业家庭解决就业困难。认真贯彻《劳动合同法》，大力发展和谐稳定的劳动关系，依法维护劳动者权益。高度重视高校毕业生就业指导工作，认真做好退役士兵和在穗就业农民工职业技能培训，鼓励自主创业和自谋职业，努力实现全市城镇净增就业人数20万人的目标。

　　积极做好社会保险、社会救助和社会福利工作。贯彻分类施保、扩大覆盖、保障基本的原则，逐步构建共享型社会保险体系。推进社会保险扩面工作，妥善解决基本养老保险历史遗留问题。调整提高企业退休人员基本养老金。将没有办理城镇职工基本医疗保险的户籍人员纳入医疗保险范围。建立和完善适合农民工、农转居人员和被征地农民的社会保障制度，继续推进农民工先行参加工伤保险，探索建立农村养老保险制度。推进社会救助体系建设，提高城镇居民最低生活保障标准和低收入困难家庭认定标准。完善城乡低保制度和基本医疗制度，实施城乡困难群众重大疾病医疗救助政策。加强流浪乞讨人员的救助管理。大力倡导社会互助，加快发展慈善事业，鼓励发展民办福利机构和义工、志愿者组织。积极发展残疾人事业和老龄事业，推进社区居家养老服务，做好老年人优待工作。

　　加快推进医疗卫生事业发展。进一步完善城市社区卫生服务体系。加强大型医院与社区卫生服务机构对口帮扶工作，逐步建立双向转诊制度。完善重大疾病防控体系，提高突发公共卫生事件应急处置能力。继续抓好中医药强市建设，推动中医药发展。深入开展爱国卫生运动，普及健康教育。加强卫生监督执法，保障公共卫生安全。加强妇幼卫生工作。稳步实施医药卫生体制改革，强化公立医院公益性质，努力为群众提供安全、有效、方便、价廉的公共卫生服务和基本医疗服务。

　　进一步完善住房保障体系。把解决群众住房问题摆上重要位置，坚持一手抓住房保障体系建设，一手抓房地产市场调控。建立健全保障型住房财政投入政策和优先供地机制，不断扩大住房保障覆盖面。加快廉租房、经济适用房建设，逐步解决符合有关规定的低收入家庭住房问题。根据房地产市场情况，合理确定商品住房用地供应规模，重点保障中低价位、中小套型普通商品住房用地供应，适度发展限价商品房，进一步改善住房供求关系和结构，抑制房价过快增长。继续推进住房分配货币化改革，完善配套政策。加强土地市场管理，推进闲置土地处置工作，坚决打击囤积土地行为，调控和维持房地产市场健康稳定持续发展。积极推进旧住宅区综合整治和危破房改造，逐步改善人民群众的居住条件和环境。进一步做好村镇、村庄规划，用三年时间帮助困难农民改造危破房。

　　保持价格总水平基本稳定。加强“菜篮子”工程基地规划建设，扶持生猪等“菜篮子”产品生产，并加强采购，切实保障主要农产品基本供给不脱销、不断档。建立和完善重要商品物资储备制度。加强市场监管，及时发布信息，确保“菜篮子”产品价格稳定。严厉查处串通涨价、囤积居奇、哄抬物价行为。

　　加快发展公共交通。完善公交地铁接驳，推进快速公交系统建设，努力缓解中心城区交通阻塞问题。大力推进公共交通站场、港湾式停靠站建设。加强城市交通信息化建设，优化交通组织管理。引入社会资金开发停车场建设，重点抓好火车东站、天河体育中心和琶洲会展中心等地下车场建设，力争今年新增5万个停车位，缓解停车难问题。

　　大力推进“平安广州”建设。继续把加强社会治安综合治理，推动社会治安进一步好转，增强人民群众的安全感摆在突出位置。保持严打高压态势，严厉打击各种黑恶势力犯罪和其他严重暴力犯罪，大力扫除“黄赌毒”等社会丑恶现象。推进城乡社区警务工作和群防群治队伍建设，完善治安视频监控系统建设。积极创建“六好”平安和谐社区。落实安全生产责任制，严格安全执法。强化对道路交通、市政设施、危险化学品、非煤矿山和特种设备等领域安全监管，严防重特大安全生产事故发生。全面完成城乡水利防灾减灾18项工程建设任务。抓好地质灾害预防工作。加强消防、防疫设施和队伍建设。认真总结今年春运工作的经验教训，进一步健全城乡突发事件应急管理体制机制，完善各种应急预案，提高政府应对复杂局面和处置突发公共事件能力。组织编制新一轮人防工程规划，做好人防应急准备。构建预防化解社会矛盾的长效机制，畅通信访渠道，加强人民调解、行政调解和司法调解的衔接配合，加强法制宣传教育和法律援助工作，健全维护群众权益机制。

　　（八）全面加强政府自身建设，进一步提高政府依法行政能力。

　　积极推进依法行政。抓紧制定和完善政府规章，严格按照法定权限和程序行使权力，提高依法管理社会事务和其他行政事务的能力。建立和完善决策项目预告制度和重大事项社会公示、社会听证制度，以及新闻发布制度。大力推进政务公开，推进决策科学化、民主化、法制化。进一步强化监督行政执法行为，规范行政执法裁量权，促进合法合理行政。自觉接受人民代表大会及其常委会的法律监督和工作监督，接受人民政协的民主监督，接受新闻舆论和社会公众监督，不断改进政府工作。充分发挥各民主党派、工商联、无党派人士和社会各界的参政议政作用，发挥工会、共青团、妇联等人民团体的桥梁和纽带作用，让人民群众更广泛地参与社会管理和公共服务。

　　大力加强政风建设。深入贯彻实施《公务员法》，建立健全行政部门绩效管理考核体系和行政过错责任追究制，规范各类行政行为。加强对公务员的教育、培训、考核、管理和监督，坚决纠正行政不作为和乱作为现象，提高政府的公信力和执行力。进一步转变工作作风，大力弘扬求真务实精神，坚决反对官僚主义、形式主义。进一步精简会议、文电，改进会风、文风，加强调查研究，把主要精力放在抓发展、抓落实和为基层、为群众服务上。

　　深入开展反腐倡廉工作。加强理想信念和廉洁从政教育，严格执行廉洁从政的各项规定，进一步规范领导干部从政行为。以确保行政权力正确运行为重点，不断深化行政监察、财政监督、审计监督和政府督查工作，完善对权力的监督制约机制。进一步加强生产要素市场建设和管理，积极拓展从源头上防治腐败的工作领域，扎实推进反腐倡廉工作。继续深入开展治理商业贿赂专项工作，努力建立健全维护群众利益的长效机制。严肃查处违法违纪案件，坚决纠正损害群众利益的不正之风。

　　各位代表，在新的起点上实现广州的新发展，是时代和全市人民赋予我们的光荣使命。展望未来，我们充满信心。让我们更加紧密地团结在以胡锦涛同志为总书记的党中央周围，在市委的正确领导下，高举中国特色社会主义伟大旗帜，坚持以邓小平理论和“三个代表”重要思想为指导，全面贯彻落实科学发展观，解放思想，开拓创新，为实现经济社会又好又快发展，让广州人民过上富裕安康的生活而努力奋斗！
